# The aesthetic triad and traditional Chinese art symbols: a theoretical framework

**DOI:** 10.3389/fpsyg.2026.1789978

**Published:** 2026-04-08

**Authors:** Wei-Na Mao

**Affiliations:** School of Art, Southeast University, Nanjing, China

**Keywords:** aesthetic triad model, cross-cultural aesthetics, neuroaesthetics, traditional Chinese art symbols, Yijing (artistic conception)

## Abstract

Neuroaesthetics has developed greatly in its understanding of aesthetic processing mechanisms in the brain, but current models are mostly based on European artistic traditions. This study aims to overcome this theoretical constraint by extending the aesthetic triad model framework that is more applicable to traditional Chinese artistic symbols. Using an integrative literature review in cognitive neuroscience, traditional Chinese aesthetic philosophy, and cross-cultural psychology, three culturally specific mechanisms are proposed, each extending one subsystem of the aesthetic triad: Embodied Calligraphic Resonance extends the perception-movement subsystem by incorporating motor simulation of brushstroke dynamics in calligraphy appreciation; Yijing Empathy extends the emotion-evaluation subsystem by capturing the holistic and transcendent emotional resonance particular to Chinese artistic conception; and Cultural Schema Activation extends the meaning-knowledge subsystem through the top-down activation of culturally specific knowledge structures. Unlike general semantic processing, these schemas are distinguished by the exceptional conceptual density of Chinese written symbols and the deep internalization of Taoist, Confucian, and Buddhist philosophical frameworks as aesthetic evaluation criteria. The new framework proposes the addition of a cultural mediation level that connects individual-level cultural variables with system-level processing, which accounts for phenomena that existing models are unable to describe. The hypotheses are formulated for each mechanism and provide specific guidance for future research. This framework advances neuroaesthetics toward a culturally pluralistic perspective, with explicit grounding in the Chinese aesthetic tradition as an initial case for non-Western theoretical extension.

## Introduction

Neuroaesthetics, as a transdisciplinary area of research that bridges cognitive neuroscience and aesthetics studies, has made significant progress in deciphering the neural basis of human aesthetics experiences over the past two decades. Neuroaesthetics tackles challenges of methodology that are common to traditional aesthetics research while providing fresh perspectives for aesthetics research. The reason for cognitive research focusing on aesthetics lies in its need to explore what lies at the essence of human cognition. Aesthetic experiences, defined as highly integrated cognitive-emotional experiences that involve a range of psychological faculties like perception, memory, emotion, and decision-making, provide a prime research subject for studying the universality of cognitive theories (Wassiliwizky and Menninghaus, 2021). Among various theoretical frameworks, the aesthetic triad model combines the perception-movement system, the emotion-evaluation system, and the meaning-knowledge system in order to describe the multi-level processes from perception to meaning. The model provides a theoretical explanation for individual differences and context dependencies in aesthetics (Vartanian and Chatterjee, 2021). The core idea of this model is that the experience of beauty cannot result from the activity of a single region of the brain or a single neural pathway, but rather it arises from the interaction of multiple functional networks of the brain. This represents an evolution of the early neural localization of beauty model of neuroaesthetics, moving toward a more complex systems-theoretical approach.

Neuroaesthetic studies have accumulated a great many methodological tools. The combination of non-invasive procedures such as functional magnetic resonance imaging, electroencephalography, transcranial magnetic stimulation, and transcranial direct current stimulation makes it possible to determine aesthetic processing activity patterns with a high degree of precision in terms of both the temporal and spatial resolution perspectives (Cattaneo, 2020). These technological developments have greatly enabled the empirical confirmation of the neural basis of each subsystem in the aesthetic triad model, such as the relationship between the ventral visual pathway and the perception-movement system, the functional link between the orbitofrontal cortex and the emotion-evaluation system, and the simultaneous neural activation of the default mode network and the meaning-knowledge system. However, it is important to note that there is a significant degree of cultural homogeneity in both samples and materials in current neuroaesthetics studies, in that most studies are done using Western classical paintings, impressionism, or abstraction, while most samples are composed of students in universities in Europe and America. The inherent limitations in this research design impose a significant cultural bias in theoretical models, while cross-cultural universality poses significant theoretical and empirical problems.

The traditional Chinese symbol system of art, with its various visual forms of calligraphy, ink painting, bronze patterns, and jade shapes, exemplifies basic differences between Eastern and Western art traditions about aesthetic principles, creative methods, and cultural semantics. Calligraphy emphasizes the process of time and its perception of vitality and charm; ink painting aims at spatial arrangement with a dialectical contrast between empty and substantive spaces and an emotional expression with an artistic conception of profound meaning; bronze design involves important ritual symbols and cosmological ideas. All these aspects constitute a special visual symbol system; thus, the aesthetic processing mechanism cannot be fully encompassed by the theoretical frameworks based on Western art experience. Recently, the study of the artificial intelligence technology in the traditional Chinese painting creation has started to disclose the special computational properties in the art aesthetics of the East. From experiments on production of landscape painting based on human and machine collaboration, aesthetic appraisal of Chinese painting involves complex and multidimensional interactions that include brushstroke flavor, compositional atmosphere, and cultural implications. They display critical conceptual distinctions from form beauty, expressiveness, and originality that dominate appraisal criteria of Western painting (Chang and Huang, 2021). From the above analysis, a key research question is derived as a pressing issue: How can we, while maintaining the key framework of explanation of the aesthetic triad model, incorporate cultural specificity variables to enable it to be applicable to non-Western art, especially regarding aesthetic processing mechanisms of traditional Chinese art symbols? The present framework is designed specifically for Chinese art traditions, while acknowledging that future cross-cultural work may explore its applicability to related East Asian aesthetic systems.

In response to the theoretical gaps and empirical requirements outlined earlier, the research aims to provide a more detailed framework of the aesthetic triad relevant to traditional symbols of Chinese art through a literature review and theoretical development. In this regard, there are three research aims: at the conceptual level, through the inclusion of traditional Chinese aesthetics such as “Qiyun” (vital resonance), “Yijing” (artistic conception), and “Shensi” (spiritual likeness), the theoretical meaning of each subsystem of the aesthetic triad model can be enhanced, thereby allowing the model to capture the special qualities of the aesthetic experience of Eastern art more adequately; at the mechanistic level, three culturally specific aesthetic processing mechanisms are proposed to be identified: Embodied Calligraphic Resonance, Yijing Empathy, and Cultural Schema Activation. Hypotheses are formulated for testing; at the methodological level, an analysis framework is outlined to develop cross-cultural neuroaesthetics research. This research aims to move the study of neuroaesthetics along its trajectory from a Western paradigm to a paradigm of cultural pluralism. Theoretical contributions of this research lie in going beyond filling a void in neuroaesthetics studies of non-Western art, to enhance an understanding of the dialectical unity of universality and particularity in human aesthetic experience. This is done through an integration of Eastern and Western approaches to aesthetics, thus providing a basis for construction of a culturally grounded theory of neuroaesthetics with explicit focus on the Chinese aesthetic tradition as an initial case. This integrative review brings together literature from three domains of knowledge: cognitive neuroscience and neuroaesthetics, traditional Chinese aesthetics, and cross-cultural psychology. In the literature research, the focus was on peer-reviewed empirical research from the past ten years (2015–2025) that highlighted neuroimaging research and cross-cultural comparisons. Theoretical contributions from traditional Chinese aesthetics that do not have specific publication dates have been included.

## Theoretical foundation and cross-cultural applicability of the aesthetic triad model

### Core components and neural mechanisms of the aesthetic triad model

The theoretical basis for the aesthetic triad model is founded upon the premise that there are three functionally differentiated yet holarchically related neural and cognitive systems. These systems each have a particular and irreducible role to play in the aesthetic processing stream and together provide the neural basis for the aesthetic experience of visual art. These systems are the perception-movement system, the first stage of aesthetic processing, that is largely concerned with the coding and representation of the visual properties of artistic stimuli such as the extraction and integration of fundamental visual attributes such as color, shape, and motion trajectory. As far as the central issue of the “neural center of beauty” in the brain during aesthetic processing is concerned, a meta-analysis of functional magnetic resonance imaging studies that focused on the neural correlates of facial versus visual artistic aesthetics has suggested that while the specific regions activated for different types of aesthetic stimuli vary, a common set of regions is activated that includes the orbitofrontal cortex, insula, and basal ganglia. This is a good indication that a stable core network is used for aesthetic processing (Hu et al., 2020). This result has significant methodological implications about the neural mechanisms of aesthetic values of traditional Chinese art symbols. Specifically, when examining the neural basis of culturally specific aesthetic experiences, it is crucial to address both the shared cross-cultural aesthetic network as well as the culture-specific regulatory mechanisms.

Another important element of the perception-movement system is the embodied simulation process, during which the observer has implicit neural activations in the motor system related to the actions involved in artwork creation or to the content expressed in the artwork. Studies that examined the neural dissociation between embodied and emotional reactions during artwork evaluation have shown that motor cortex activation can be distinguished from the activation of regions involved in emotional processing. This result indicates that the embodied simulation is an aesthetic processing pathway that is independent of emotional appraisal, possibly facilitating the connection between the observer and the artistic intention or the action expressed in the artwork through the mirror neuron system (Finisguerra et al., 2021). This process is of particular interest for the explanation of the aesthetic appraisal of Chinese calligraphy, where the appraisal not only requires the viewing of the static character but also the dynamic movement of the brush strokes expressed by the writer. This Embodied Calligraphic Resonance may be the specific neural footprint of the traditional Chinese art aesthetic.

The emotion-evaluation system plays a pivotal role in the aesthetic triad model, which converts perceptual information into a subjective aesthetic evaluation. This process involves the coordinated operation of several subsystems, such as the reward system, emotional processing network, and self-processing system. The default mode network, which plays a pivotal role in the emotion-evaluation system, plays a key part in the process of aesthetic experience. Neuroimaging studies focusing on the dynamic process of aesthetic experience show that when participants experience intense aesthetic emotion, their default mode network activity follows specific dynamic patterns in accordance with the subjective experience of the aesthetic process (Belfi et al., 2019). Further studies have also revealed that the beauty associated with default mode network activation follows a generalized pattern in terms of visual domains. Whether it is natural scenes, buildings, or artwork, default mode network activation is always predictive of individual scores of aesthetic preferences, suggesting that the emotion-evaluation system processes an abstract signal of aesthetic values (Vessel et al., 2019). The meaning-knowledge system is responsible for controlling the location of aesthetic objects in a semantic framework to enable understanding. The realization of the meaning of the meaning-knowledge system relies on the extraction and integration of cognitive resources such as artistic history information in long-term memory. This process involves the semantic processing area of the temporal lobe and the executive control network of the prefrontal area. These three systems do not process information in a sequential manner. Instead, they enable information exchange through dynamic connectivity between large-scale brain networks. The aesthetic experience is a result of a multi-system cooperative process.

### Current state and limitations of cross-cultural research on the aesthetic triad

Even if the aesthetic triad model provides a strong basis for explaining the underlying neural mechanisms of aesthetic experiences, the model, together with the body of empirical research, has a prominent blind spot in terms of cross-cultural differences. Modern studies are dominated by Western traditions in terms of participants, stimuli, and underlying theories. This not only leads to a limited generalizability of the findings, but it may also conceal the unique aesthetic processing mechanisms in non-Western cultures. The systematic investigation of the role of professional knowledge and cultural factors in the appreciation of visual artworks has shown that the cultural factor has a strong regulatory effect on all three aspects of the aesthetic triad. Among them, it is the meaning-knowledge system that is most obviously and deeply affected by cultural influences, while the cultural regulatory role of the perception-movement system is much more subtle, as it is primarily reflected in attention bias and processing priority of specific visual details (Darda and Cross, 2022). These results form a preliminary theoretical basis for explaining cultural specifics of aesthetic experiences, but existing studies are dominated by using Western art works as stimulus material in cross-cultural comparisons, while there are no systematic analyses of aesthetic processing specifics of non-Western art traditions.

The traditional Chinese aesthetic system, based on a unique philosophical basis and aesthetic categories, has developed a theoretical discourse that is quite distinct from that of Western aesthetics. Specifically, Yijing describes the overall aesthetic essence of the piece of art—namely, the smooth integration of the setting, the feeling, and the idea beyond the scene depicted. Shensi, on the other hand, describes the viewer’s power of imagination which enables access to this aesthetic essence. Yijing and Shensi are the conceptual basis for the Yijing Empathy mechanism discussed in this study. The traditional Chinese aesthetic notions of “Qiyun” (vital resonance), “Yijing” (artistic conception), and “Shensi” (spiritual likeness) cannot be simply incorporated within the dominant aesthetics of the aesthetic triad. The research on traditional Chinese aesthetics’ methodology of artistic aesthetics reveals that Chinese aesthetics attach primary importance to spiritual resonance between artist and viewer, internal unity of artwork and nature, and pursuit of artistic conception beyond representation. The aesthetic principles imply a cognitive neural process which may diverge in some fundamental aspects from the Western aesthetic processing mechanism (He, 2022). However, the task of incorporating the Chinese aesthetic ideas and hypotheses in the framework of aesthetic triad model has remained unexplored in the field of study.

**Figure 1:**
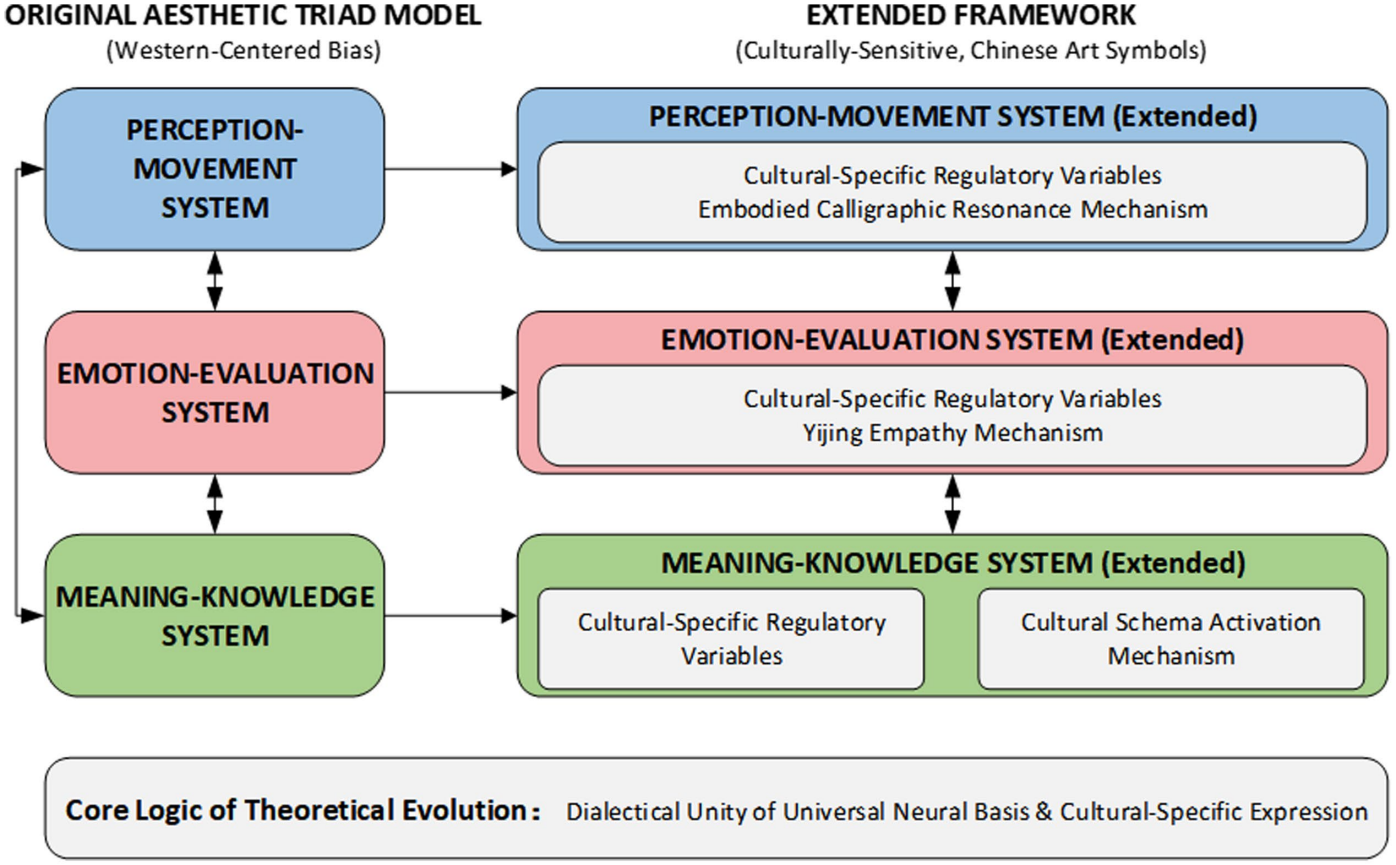
From aesthetic triad to extended framework: theoretical evolution.

To clearly illustrate the theoretical logic of the aesthetic triad model evolving into the framework of cultural sensitivity, Figure 1 presents the overall context and core elements of this theoretical development, serving to visualize the logical pathway from the original aesthetic triad to the culturally-extended framework.

As shown in Figure 1, the aesthetic triad model’s core systems must be complemented by theoretical notions of regulatory variables specific to culture when dealing with the symbols of traditional Chinese artworks. The perception-movement system must include the Embodied Calligraphic Resonance process to match the aesthetic processing abilities of dynamic artworks, such as calligraphy; while the emotion-evaluation system must include the Yijing Empathy idea to reflect the unique mechanisms of evoking emotions involved in Chinese artworks; and the meaning-knowledge system must activate the Cultural Schema Activation mechanism in order to explain the semantic processing of traditional symbols. The central argument of the theoretical development depends on the recognition of the dialectical unity of the universal neurological basis of aesthetic experience and the culturally specific modes of expression.

## Analysis of the aesthetic characteristics of traditional Chinese art symbols

### Perception-movement dimension: visual coding and embodied characteristics of traditional Chinese art

The symbol of Chinese art represents the specific rules for the visual encoding of the perception and movement dimension, which are very different from the Western art symbol. The reason for the difference lies in the contrast between Eastern and Western art mediums and aesthetic concepts. Calligraphy, which represents the essence of Chinese art, involves an aesthetic process that includes the perception and simulation of the brush movement trajectory. When viewers interact with calligraphy artworks, they not only passively receive the visual form information but also unconsciously recreate the motor actions of the calligraphy writer via the mirror neuron system. The functional magnetic resonance imaging experiment on the functional role of action observation in calligraphy aesthetics reveals that the viewers show considerable activation in the motor areas and the parietal cortex when they observe calligraphy, and the activation is positively related to the amount of calligraphy training the viewers have, which suggests that the aesthetic preference formation for calligraphy is related to the resonance between the viewers’ experiences and the stimuli they receive (He et al., 2021). This result shows the embodied aspect of calligraphy aesthetics, suggesting that aesthetic evaluation is not only a visual analytical process but also closely linked to the relationship between perception and action of the body movement system.

The neural structures that participate in the aesthetic experience of calligraphy not only include the activation of specific brain regions but also the dynamic alteration of the brain networks. Through the use of brain network analysis techniques, the study shows that the strength of the functional connectivity between the visual networks, the motor networks, and the default mode networks significantly changes when the aesthetic experience of calligraphy takes place (Li et al., 2022). Ink painting, a traditional form of Chinese art, displays the characteristics of visual encoding, in which the principles of spatial organization are focused on the dialectical relationship of empty and full space, the scattering of perspectives, and the stratified variation of ink and brush strokes. The modeling of the visual complexity of ink painting suggests that the visual complexity of Chinese ink painting requires the non-linear integration of the multi-dimensional parameter of ink color distribution, spatial density, and the edge features. Its complexity perception mode is significantly different from that of Western oil painting, which is more dependent on parameters such as color saturation and light–dark contrast (Fan et al., 2022). The mediated theory approach to embodied cognition provides a more macro-explanatory framework for explaining these differences. This is because the mediated theory suggests that the embodied dimension of aesthetic experience is deeply affected by the media environment within which it is situated. In this system of understanding, the experience of embodiment refers to the automatic motor resonance that is triggered when viewing art, which is the engine for Embodied Calligraphic Resonance. Enactive experience refers to the viewing habits of the spectators based on their culture, through which they create meaning for the artwork rather than simply consuming it. Together, these two dimensions define the perception-movement subsystem’s culturally extended role: embodied simulation provides the sensorimotor scaffold, and enactive enculturation determines which features of an artwork are perceptually foregrounded (Fingerhut, 2021).

### Emotional-evaluation dimension: the emotional arousing mechanism of traditional Chinese art

The emotion-evaluation dimension in traditional Chinese art is a special kind of categorical framework and mechanism of arousal. The most essential feature is the focus on the implicitness, completeness, and transcendence of emotions, as opposed to explicit identification and definition of emotions in the aesthetic traditions of the West. *Yijing* (artistic conception) is the central concept underlying emotional arousal in Chinese art. Unlike a discrete emotional state, it denotes a holistic aesthetic quality of the artwork itself—the fusion of scene, emotion, and transcendent meaning—that viewers access through *Shensi* (神思), the imaginative projection faculty theorized in classical Chinese aesthetics. It is through this *Shensi*-mediated engagement that the emotional resonance characteristic of Yijing Empathy arises (Jing, 2023). The process of emotional stimulation has important conceptual differences compared to a two-dimensional approach to emotions based on valence and arousal levels in Western aesthetics. It also requires a more sensitive approach to create a suitable theoretical platform for understanding.

Affective evaluation of calligraphic art has developed a sophisticated system of categories for the style-emotion of calligraphy, such as “vitality,” “elegance,” “authenticity,” and “refinement.” These categories not only allude to the characteristics of the art but also suggest evaluations of the personality and character of the calligrapher. In cross-cultural research on the evaluation of calligraphic aesthetics, it has been discovered that Chinese and Western evaluators tend to use different cognitive strategies when evaluating the same work of art. Chinese tend to focus on the overall ambiance or spiritual quality of the work, while Westerners tend to focus on more structural elements, such as balance and complexity (Xu and Shen, 2023). This cross-cultural difference can be further evidenced by the cultural matching effect, which indicates that when the cultural context of the artwork matches the viewer’s cultural identity, the strength of the emotional tie and the passion for aesthetic evaluation are significantly increased. This phenomenon is more evident in traditional art form (Ho et al., 2023). The physiological basis for the arousal of aesthetic emotions should not be ignored. Studies on the correlation between bodily sensations and aesthetic experiences in artworks imply that aesthetic experiences are not purely cognitive but have a strong basis in the variation of bodily sensation states. Different artwork genres can stimulate different patterns of bodily sensations, which could be the basis for aesthetic evaluation (Nummenmaa and Hari, 2023). With respect to the neural correlates of Yijing Empathy, the focus is not on mind-wandering and autobiographical memory recall per se, but on the continuous functional connectivity between the medial prefrontal cortex and the posterior cingulate cortex. This type of connectivity is associated with self-referential imaginative engagement and is related to peak aesthetic experiences in all visual domains (Vessel et al., 2019; Belfi et al., 2019).

### Meaning - knowledge dimension: cultural semantic processing of traditional Chinese art

The dimension of meaning-knowledge gains special significance in the aesthetic processing of traditional Chinese artistic symbols. This is because of the significant importance accorded to the symbolic meaning of signs in traditional Chinese art discourse, along with its close connection to artistic evaluation and cultural knowledge. Traditional Chinese artistic symbols are normally composed of a complex semantic structure that varies from superficial aesthetic properties to middle-level symbolic meanings to deep-level philosophical notions. For example, traditional Chinese symbols like the dragon sign are composed not only of aesthetic properties like its flowing shape but are additionally replete with diverse meanings like imperial power, harmony of the cosmos, and transformation of yin and yang. The degree of understanding of these meaning categories will have a direct effect on the level of the aesthetic experience. Based on the “Six Principles Theory” of Xie He, the study of the computational approach for Chinese paintings attempts to express the aesthetic categories of the traditional Chinese painting theory in the form of operable and quantifiable elements. The notions of ‘vitality and liveliness’ (*qiyun shengdong*, 氣韻生動—the first of Xie He’s Six Principles, referring to the spiritual animation and rhythmic energy that animates a work) and the ‘bone method of brushwork’ (*gufa yongbi*, 骨法用筆—the structural force and expressive power conveyed through brushwork technique) are broken down into quantifiable indicators such as brushwork dynamic characteristics, structural tension, and breathing (Zhang et al., 2024).

The regulatory role of cultural knowledge reserves in forming the understanding of the aesthetics of traditional Chinese art can be seen in several aspects, such as the ability to identify the symbolic meaning, to interpret allusions, and to understand the philosophical context. Internal perception processing is a link between the physical and emotional assessment and is involved in artistic meaning formation. Neuroimaging studies on the basis of internal perception in artistic emotion assessment have shown that the extent to which the key areas involved in internal perception processing, such as the insula, are activated during aesthetic assessment is positively related to the extent to which the artwork is significant to the individual (Ardizzi et al., 2021). The philosophical traditions of Taoism, Confucianism, and Zen Buddhism have provided a deep cognitive system for the assessment of Chinese art. Unlike general semantic schema activation found across cultures, the cultural schemas operative in Chinese art appreciation are distinguished by two features: the exceptionally high conceptual density of Chinese written symbols, in which a single character may compress cosmological, historical, and affective meanings simultaneously, and the deep internalization of Taoist, Confucian, and Buddhist philosophical frameworks as aesthetic evaluation criteria rather than mere background knowledge.

**Figure 2:**
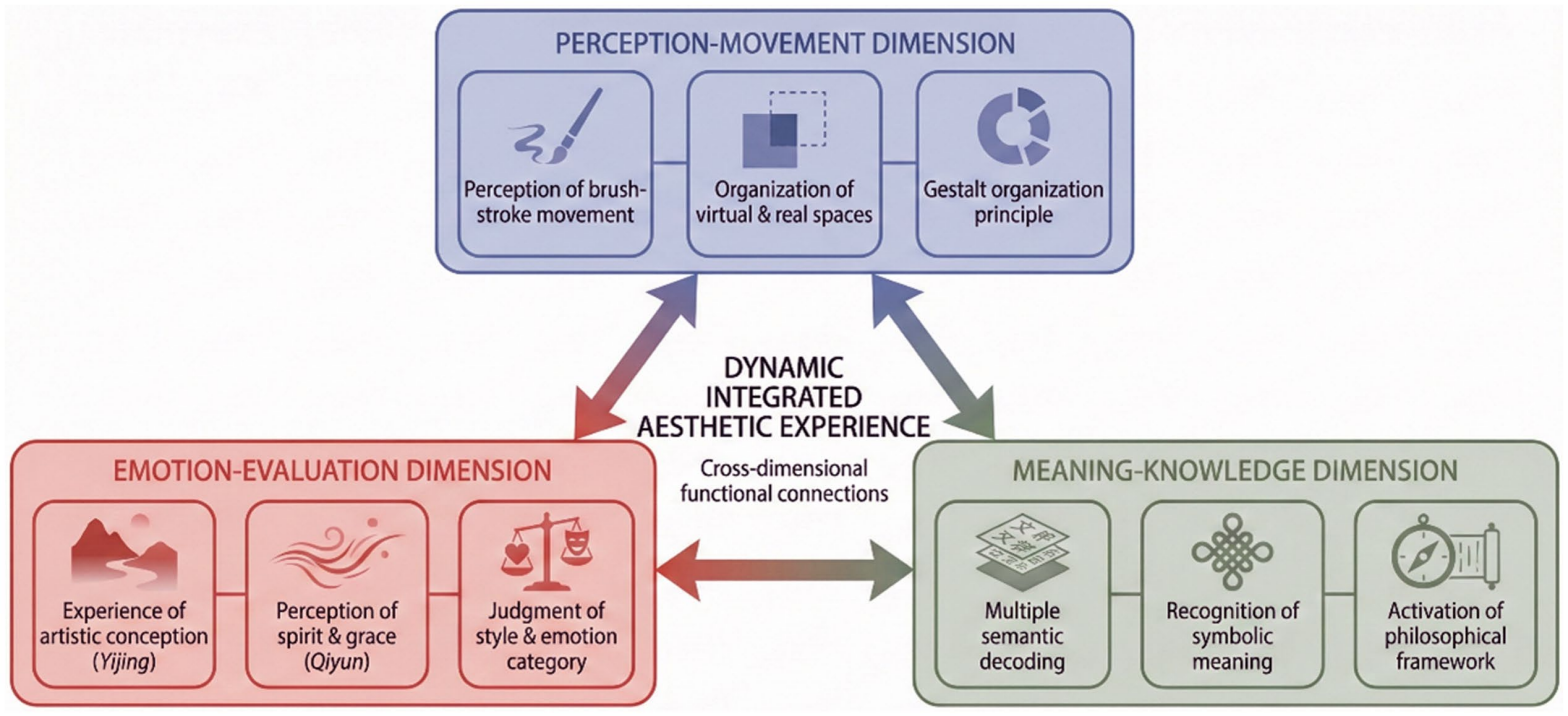
Mapping matrix of aesthetic features of Chinese traditional art symbols onto the three dimensions.

To present the specific mapping relationship of the aesthetic characteristics of traditional Chinese art symbols on the three dimensions of the aesthetic triad, Figure 2 provides an integrated analytical matrix that operationalizes how specific aesthetic features of traditional Chinese art map onto each dimension, thereby bridging theoretical constructs with empirically observable indicators.

As illustrated in Figure 2, the aesthetic features of the symbols of traditional Chinese art are mainly presented in the perception-movement dimension, including the perception of brush stroke movements, the allocation of virtual and real space, and the Gestalt organizational principle, as well as the experience of artistic conception, the perception of spirit and grace, and the evaluation of style and emotion categories in the emotion-evaluation dimension; in the meaning-knowledge dimension, the processes include multi-level semantic decoding, the perception of symbolic meaning, and the activation of philosophical frameworks, among other cognitive processes. The three dimensions above are not separate but rather an integrated aesthetic experience through cross-functional interconnections among the dimensions. This relationship between the dimensions offers an empirical basis for the development of the theoretical framework.

## Construction of the extensibility theoretical framework

### The proposal of culture-specific aesthetic processing mechanism

Based on the systematic analysis of the aesthetic attributes of traditional Chinese art symbols discussed earlier, this research postulates that three culture-specific aesthetic processing mechanisms need to be employed to fill the theoretical void in the original aesthetic triad model when dealing with the aesthetic experience of non-Western art. The first one is Embodied Calligraphic Resonance, which operates primarily in art forms with prominent movement trajectories—most paradigmatically calligraphy—and is less directly applicable to static or non-gestural forms such as ritual bronzes or jade carvings, whose aesthetic processing is more substantially governed by Yijing Empathy and Cultural Schema Activation. In this case, the observer uses the mirror neuron system to implicitly replicate the movement of the creator of the art to induce neural activity related to the writing process. The major premise for this mechanism is that the acquisition of the aesthetic preferences for calligraphy is not only based on the analysis of the static form but is rather based on the level of action resonance between the individual writing experience and the brush stroke features of the artwork. Those individuals who have had training in calligraphy are better positioned to decipher the action features that are implicit in the artwork. This process is supported by empirical evidence from neuroimaging research. It was found that the viewing of the action of calligraphic creation significantly increases the preference for the observed art, and the motor cortex was positively correlated with the individual’s experience in the art of calligraphy (He et al., 2021). Furthermore, research on the motor reaction to artworks has shown that the process of motor simulation is an independent route for aesthetic processing that does not involve emotional appraisal and that a link exists between motor simulation and empathy (Finisguerra et al., 2021).

Yijing Empathy is the second mechanism and focuses on the way of emotional elicitation unique to traditional Chinese art. It argues that viewers access the artistic conception space constituted by the works via imaginative projection and achieve a deep level of emotional resonance with the works and their authors. Compared with the discrete emotion perception mechanism of empathy found in Western aesthetics, Yijing Empathy values the wholeness and transcendental nature of emotional experience. This process requires the viewers’ ability to understand implicit expressions such as “the meaning beyond words” and “the image beyond the image.” Cultural Schema Activation is the third mechanism and explains viewers’ activation of their own cultural knowledge structures from long-term memory when interpreting traditional Chinese art symbols. These schemas are the symbolic meaning system, the philosophical concept system, and the aesthetic judgment system. The cultural schemas govern the entire aesthetic perception process. The strength of the schemas’ activation can be empirically measured through the accuracy and speed at which the symbols are identified and the areas where the viewer’s gaze lingers on the meaningful parts of the image. Such measures are the behavioral correlates and are therefore empirically testable.

These three processes are not mutually independent; instead, a dynamic functional link is created through aesthetic processing. Yijing Empathy is based on Embodied Calligraphic Resonance through sensations in the body, while Cultural Schema Activation provides a schema for interpreting these first two processes. It is important to note that, while schema activation is a general cognitive process, the schemas in this case are culturally specific in their contents and evaluations. This distinguishes CSA from general semantic processes and relates it to the culturally extended role of the meaning-knowledge system.

To clearly present the conceptual definitions, theoretical origins, and empirical testable research hypotheses of the three cultural-specific aesthetic mechanisms, Table 1 provides a systematic integration and summary.

**Table 1:** Definitions, theoretical foundations, and testable propositions of culture-specific aesthetic mechanisms.

Mechanism	Definition	Theoretical sources	Testable propositions
Embodied calligraphic resonance	When viewers appreciate calligraphy and other art forms with obvious movement trajectory characteristics, they implicitly simulate the creator’s body movements through the mirror neuron system, generating neural activation patterns corresponding to writing behavior at the motor system level. This mechanism is primarily operative in movement-salient art forms; for static artifacts such as ritual bronzes or jade carvings, aesthetic processing is more substantially governed by Yijing Empathy and Cultural Schema Activation.	Mirror neuron theory; Embodied cognition theory; Action observation research in calligraphy aesthetics (He et al., 2021; Finisguerra et al., 2021)	H1: Viewers with calligraphy training experience will show stronger motor cortex activation (measured by functional magnetic resonance imaging) when appreciating calligraphy works compared to untrained viewers. H2: The degree of motor cortex activation will be positively correlated with aesthetic preference ratings.
Yijing empathy	Viewers enter the artistic conception space created by artworks through imaginative projection, achieving deep emotional resonance with the works and their creators. This mechanism emphasizes the wholeness, ambiguity, and transcendence of emotional experiences rather than discrete emotion recognition.	Traditional Chinese Yijing aesthetics theory; Embodied emotion theory; Cross-cultural empathy research (Jing, 2023; Nummenmaa and Hari, 2023)	H3: Chinese viewers will report stronger emotional resonance and longer viewing duration for ink-wash paintings with profound artistic conception compared to Western viewers. H4: Bodily sensation mapping patterns will differ significantly between Chinese and Western viewers when viewing the same traditional artwork.
Cultural schema activation	When understanding traditional Chinese art symbols, viewers automatically invoke cultural knowledge structures stored in long-term memory, including symbolic meaning systems, philosophical concept frameworks, and aesthetic evaluation standards. These schemas function as cognitive filters shaping perception, evaluation, and meaning construction.	Schema theory; Cultural psychology; Semantic processing research (Darda and Cross, 2022; Zhang et al., 2024)	H5: Viewers with rich cultural knowledge reserves will show faster reaction times and higher accuracy in symbolic meaning recognition tasks. H6: Eye-tracking data will reveal that culturally knowledgeable viewers allocate more fixations to semantically significant areas of traditional artworks.

As shown in Table 1, each of these three mechanisms corresponds to each of the three basic dimensions of the aesthetic triad. Embodied Calligraphic Resonance generalizes the theoretical implications of the perception-movement subsystem; Yijing Empathy generalizes the theoretical implications of the emotion-evaluation subsystem; and Cultural Schema Activation further enhances the cultural sensitivity of the meaning-knowledge subsystem. In each case, operationalizable hypotheses are formulated that can be tested by functional neuroimaging techniques, eye tracking methods, or behavioral studies, which provide clear theoretical guidance for future empirical research.

In order to clarify the translation process from aesthetic concept to cognitive neuroscience, Table 2 provides the three-level mapping for both the culture-specific mechanism, with the origin from aesthetic philosophy, the psychological processing hypothesis, and the proposed neural mechanism.

**Table 2:** Three-level conceptual mapping of culture-specific aesthetic mechanisms.

Mechanism	Philosophical-aesthetic level	Psychological processing level	Neural implementation level
Embodied calligraphic resonance	Brushwork aesthetics emphasizing “bone method” (gufa) and dynamic vitality in stroke execution	Implicit motor simulation of writing movements during visual appreciation; action-perception coupling modulated by viewer’s motor expertise	Mirror neuron system activation; premotor and parietal cortex engagement; corticospinal excitability changes
Yijing empathy	Artistic conception (Yijing) as holistic fusion of emotion, scene, and transcendent meaning; “meaning beyond words”	Imaginative projection into artwork’s atmospheric space; global emotional resonance rather than discrete emotion recognition	DMN involvement, specifically sustained coupling between medial prefrontal cortex (imaginative projection) and posterior cingulate cortex (contemplative engagement); functionally dissociable from DMN-mediated mind-wandering by its artwork-anchored, *Shensi*-driven character
Cultural schema activation	Symbolic meaning systems rooted in Taoist, Confucian, and Buddhist philosophical traditions	Top-down activation of culturally-acquired knowledge structures during symbol interpretation; schema-guided attention allocation	Temporal lobe semantic areas; prefrontal executive control network; modulated N400 amplitude in Event-Related Potential studies

As shown in Table 2, the three-level mapping offers conceptual insight into the meaning of the mechanisms in traditional aesthetics, the process from a psychological perspective, and the possibility of implementation in neural substrates. This organization provides a clear conceptual pathway from philosophical constructs to the generation of empirically verifiable neuroscientific theories. It must be noted that the level of neural implementation contains theoretical predictions rather than verifiable causal associations. The neural substrates outlined have been informed by existing neuroimaging literature related to other processes and serve as hypothetical constructs for future empirical testing. Empirical validation of these neural pathways will require experimental testing using functional neuroimaging techniques.

### Integrated model of the extended framework of the three-component aesthetic system of traditional Chinese art symbols

Based on the explicit description of the three culturally specific mechanisms of aesthetic processing, this study proposes an integrated model within the expanded aesthetic triad of traditional Chinese art symbols. This model includes a cultural adjustment level while maintaining the same basic structure as the original aesthetic triad, and it explicitly describes the regulatory effect of cultural factors on each dimension of aesthetic processing. In the extended triadic model, the patterns of interaction become more complex. The Embodied Calligraphic Resonance involves the ability of the output of the perception-movement system to index the encoding of the formal aspects and to establish a direct link with the body-sensation channel of the emotion-evaluation system. The Yijing Empathy involves the establishment of a bidirectional functional loop between the emotion/evaluation system and the meaning-knowledge system, where the development of emotional experience is linked to the activation of cultural knowledge, and the enhancement of meaning comprehension is related to the enhancement of the intensity of emotional resonance. Cultural Schema Activation functions as a top-down regulatory component, but its relationship with the other two mechanisms is bidirectional rather than unidirectional: while CSA shapes the interpretive frame for ECR and Yijing Empathy, the bodily and emotional signals generated by those mechanisms in turn modulate the salience and accessibility of culturally stored schemas, forming a dynamic feedback loop across the three subsystems. Neuroscientific studies on the underlying neural bases of aesthetic experience related to visual arts as well as music provide a vital reference point to grasp the differences in the processing of aesthetic experience between the various art forms. The experiment reveals the existence of a difference between the neural implementation pathways of aesthetic experience related to the various art forms, despite the use of a common aesthetic network (Luo et al., 2025).

The introduction of the cultural mediation level is the key theoretical advance within the expanded model. These factors can be quantified using standardized psychological instruments. The level of cultural identity can be rated using the Multigroup Ethnic Identity Measure and similar instruments, while the level of aesthetic training can be rated based on the number of years the individual has practiced and the level of proficiency in standard art recognition tests. Degree of philosophical concept internalization may be approximated through culturally specific semantic priming paradigms or knowledge-based questionnaires. Together they regulate the intensity and style of activation of the three culture-specific processes. The extended model has a definite predictive role. In the perception-movement dimension, Chinese viewers with calligraphy experience are expected to show greater motor cortex activity and aesthetic appreciation when exposed to calligraphy artworks. In the emotion-evaluation dimension, viewers are expected to show greater physical sensation mapping and longer-term emotional experience when exposed to deep ink-wash artworks. In the meaning-knowledge dimension, viewers with a heavy cultural knowledge reservoir are expected to perform the processing of the semantic meaning of traditional symbols more effectively and thoroughly. The expanded framework is a triple-fold relation of inheritance, expansion, and dialogic engagement with the original triadic model of aesthetics. It is necessary to understand that expertise accounts for part of the explained variance of the phenomena targeted by the proposed hypotheses, especially H1, H3, and H5. Our model does not contradict this. Rather, it argues that cultural specificity operates through channels that are only partially and not fully accountable in terms of the effects of expertise. Specifically, ECR captures motor resonance patterns that are culturally scaffolded beyond mere technical training, Yijing Empathy involves holistic emotional orientations rooted in philosophical worldviews rather than skill acquisition, and CSA activates symbol systems whose conceptual density is culturally unique. Disentangling expertise from cultural specificity is therefore a key methodological priority for future empirical work using the proposed framework (Vartanian and Chatterjee, 2021; Darda and Cross, 2022).

**Figure 3:**
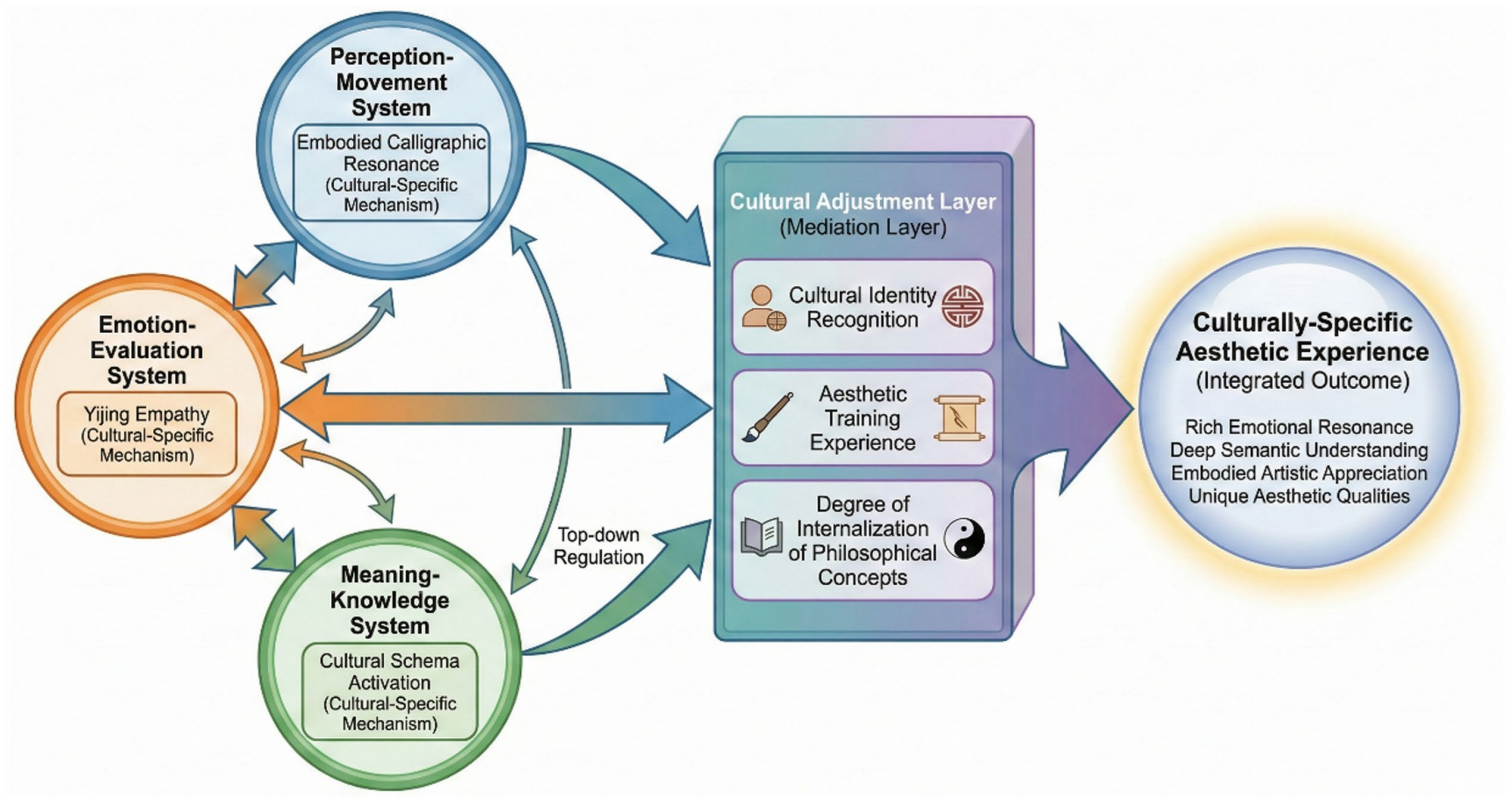
Extended aesthetic triad framework for Chinese traditional art symbols.

To visually illustrate the relationship between the overall architecture of the extended framework and its core elements, Figure 3 presents the complete model of the extended framework, synthesizing the preceding analyses into an integrated theoretical architecture that specifies both the structural relationships among components and the directional flow of aesthetic processing.

As shown in Figure 3, the extended framework is based on the triadic core of the original aesthetic model. Culturally specific mechanisms are incorporated within each subsystem, and through the cultural adjustment level, individual-level variables are linked with the processing mechanisms on a system level. The result is a culturally specific aesthetic experience. The extended framework offers a theoretical tool for explaining aesthetic processing of traditional Chinese art symbols and a scalable analytical framework for cross-cultural neuroaesthetics research.

## Discussion

The expanded aesthetic triad of traditional Chinese art symbols developed within this study has a significant theoretical role in providing a systematic theoretical contribution to the development of cross-cultural studies of neuroaesthetics. One of its most significant values is that it challenges the theoretical momentum that has been traditionally grounded in Western art experiences, providing a fresh theoretical route to decode aesthetic processing mechanisms in a non-Western cultural setting. The aesthetic processing mechanisms of Embodied Calligraphic Resonance, Yijing Empathy, and Cultural Schema Activation, which are specific to each culture, that are postulated within the expanded aesthetic triad, have created a fruitful theoretical dialogue with the empirical data of recent cross-cultural aesthetic studies. From the cross-cultural comparison study on aesthetic preference and inferential abilities among the Chinese and German groups, it can be seen that although there is cross-cultural universality for some fundamental aesthetic dimensions, cultural differences have a more significant regulatory effect on the higher-level stages of processing, where the interpretation of cultural symbols and the level of emotional intensity come into play (Miller et al., 2025). This finding offers proof for the existence of the cultural regulatory layer in the theoretical framework, suggesting that universality and cultural specificity are not necessarily contradictory but appear instead as different expressions at different stages. Further evidence of the significance of the cultural matching effect in art appreciation among the younger generation can also be observed through the examination of the research findings, which clearly show that there is a marked augmentation of enthusiasm and emotional engagement with aesthetic evaluation if the culture carried through the artwork is matched with the cultural identity of the appreciator (Szubielska et al., 2025). These findings clearly support the central tenets of the extended framework model of the Cultural Schema Activation mechanism, which states that the culture knowledge structure acts as a cognitive filter that shapes the entire process of aesthetic perception and meaning construction. One of the important differences distinguishes an extended framework from traditional cross-cultural theories. Currently, there is a descriptive bias in the existing literature, which simply records the presence of intercultural differences without specifying their mechanisms (Darda and Cross, 2022; Miller et al., 2025). In contrast to existing theories, an extended framework promotes mechanism specification based upon three processing paths to produce specific neural hypotheses. In addition, existing theories consider culture to be a moderating variable. In contrast to existing theories, an extended framework considers culture to be operating through multiple mechanisms. This allows the explanation of phenomena which previous theories can only address in a very superficial way. This is the case when it comes to the improvement of aesthetic perception due to calligraphy training and the experience of Yijing-based events. The processing of traditional symbols is linked to philosophical knowledge structures which are absent in the Western tradition.

The theoretical progress of the extended framework is also reflected in its substantive contribution to the long-standing debate between ‘aesthetic universality versus cultural specificity’, not by resolving it definitively, but by demonstrating that universality and cultural specificity are complementary expressions operating at different levels of aesthetic processing rather than mutually exclusive positions. The empirical study of the way cultural frameworks influence aesthetic assessments of abstract and concrete artworks has shown that the cultural contextual data provided with the artwork can significantly affect the assessment strategies and aesthetic preferences of viewers. This is especially true with regard to concrete artworks (Darda et al., 2023). The expanded framework successfully combines universality and specificity by including mechanisms of cultural specificity in each of the three systems of the original aesthetic triad, as well as adding a layer of cultural adjustment to mediate between individual variables and system-level processing. This study also pushes forward the interpretation of traditional Chinese aesthetics in terms of cognitive science. Traditional concepts of aesthetics, such as “Yijing” and “Qiyun,” are assigned operational definitions and testable hypotheses in terms of theoretical mapping to neural and cognitive processes. This cross-disciplinary recasting of concepts enables entirely new paths of communication between Eastern and Western aesthetics.

The present framework carries clear forward-looking relevance at the level of theoretical development. However, in the context of a purely theoretical study, the correctness of the general framework must be validated in systematic empirical work. The cross-cultural study of the fixation time of eye movement in painting representations has significant methodological references for future empirical work. The differences in fixation behavior of Easterners and Westerners in the perception of paintings are considerable, and these differences already appear in the initial phase of the fixation sequence (Trawiński et al., 2023). The combination of eye-tracking technology and functional neuroimaging will provide strong technological support for the validation of the Embodied Calligraphic Resonance mechanism and the Cultural Schema Activation mechanism. Further studies on cross-cultural painting-viewing have revealed that culture not only affects the results of aesthetic evaluation but also deeply shapes the strategies and paths of visual exploration (Trawiński et al., 2024). The application of machine learning methods to aesthetic judgment prediction research represents a new approach to the validation of the extended model. Analysis on the predictive elements of creative aesthetic judgment of Western art has found symbolism, emotionality, and imagination to be the key predictive elements of aesthetic judgment (Spee et al., 2023). The establishment of a model of aesthetic judgment of visual art that encompasses the representation of content and attributes of perception of form is a further verification of the methodology of multi-dimensional analysis (Spee et al., 2024). The machine learning comparative study of cross-cultural aesthetic judgment in Japanese and German cultures reveals characteristic patterns in the weighting of aesthetic attributes in each culture (Mikuni et al., 2024). Future studies will utilize the improvements in methodology discussed above to develop cross-cultural comparison studies on traditional Chinese art symbols with the objective of systematically examining the theoretical assumptions proposed by the proposed framework. The application level of the expanded framework has wide prospects, which include digital preservation and dissemination of traditional artwork in digital humanities studies, development of cross-cultural aesthetic skills for artwork education, and interpretation of traditional symbols for cultural heritage conservation. Each of these has potential for being developed and guided through the theoretical outlook that is presented through the framework.

The present framework is specifically grounded in the Chinese aesthetic tradition and should not be assumed to transfer without modification to neighboring East Asian contexts. Although Japanese and Korean aesthetics share certain philosophical influences with Chinese art—such as Zen Buddhism and ink wash painting—their core aesthetic concepts, such as Japanese *wabi-sabi* and *ma*, involve distinct spatial and emotional orientations that would require separate theoretical specification. The framework is therefore best understood as a culturally grounded model for Chinese art that may serve as a methodological template for developing parallel frameworks in other East Asian traditions.

Several limitations of the present framework should be acknowledged. First, it should be pointed out that the ECR mechanism is primarily calligraphy-centric, and it is unclear how well it will translate to other movement-heavy forms of art. Second, it should be pointed out that these three mechanisms are primarily theoretical constructs, and their neurological basis is inferred from other studies rather than having been directly measured or observed. Causal evidence for these mechanisms will need to be found in neuroimaging and behavioral studies using traditional Chinese art. Third, the cultural mediation layer variables, though operationally specified, require validation as reliable and culturally sensitive psychometric instruments. Fourth, the framework does not yet account for individual variation within Chinese viewers, including generational differences in philosophical knowledge and varying degrees of cultural identification among diaspora populations. These limitations define a clear empirical agenda for future research.

## Conclusion

This research is significant to the area of neuroaesthetics because it proposes and develops the aesthetic triad theory with the addition of three culture-specific models: Embodied Calligraphic Resonance, Yijing Empathy, and Cultural Schema Activation. These models are further supported by the addition of the culture mediation layer that bridges the individual-level and system-level aesthetic process. It is the integration and interrelation between aesthetic universals and culture-specificity that is the primary theoretical contribution to this research. It is shown that universals and specificity are complementary and not opposing factors.

The extended theory yields a set of six hypotheses that can be empirically tested for validation with functional neuroimaging and eye-tracking techniques. The implications of such a theoretical tool can be applied in digital humanities projects such as traditional artwork preservation and cross-cultural art education and conservation. While it is necessary that these hypotheses be validated from a scientific point of view, it can be said that such a theoretical basis for neuroaesthetics brings about a much-needed culturally inclusive approach toward a field that seeks to understand the complexities of human aesthetics.
